# Aberrant Patterns of Sensory-Evoked Activity in the Olfactory Bulb of LRRK2 Knockout Mice

**DOI:** 10.3390/cells10113212

**Published:** 2021-11-17

**Authors:** Andrea Maset, Marco Albanesi, Antonio di Soccio, Martina Canova, Marco dal Maschio, Claudia Lodovichi

**Affiliations:** 1 Veneto Institute of Molecular Medicine, Via Orus 2, 35129 Padova, Italy; andrea.maset90@gmail.com (A.M.); marco.albanesi@studenti.unipd.it (M.A.); disoccio.antonio@gmail.com (A.d.S.); 2 Padova Neuroscience Center (PNC), Università degli Studi di Padova Via Orus 2, 35129 Padova, Italy; martina.canova@studenti.units.it (M.C.); marco.dalmaschio@unipd.it (M.d.M.); 3 Department of Biomedical Sciences-UNIPD, Università degli Studi di Padova, Via U. Bassi 58B, 35121 Padova, Italy; 4 Institute of Neuroscience-CNR, Viale G. Colombo 3, 35121 Padova, Italy

**Keywords:** LRRK2, olfactory alterations, Parkinson’s disease

## Abstract

The LRRK2 gene is the major genetic determinant of familiar Parkinson’s disease (PD). Leucine-rich repeat kinase 2 (LRRK2) is a multidomain protein involved in several intracellular signaling pathways. A wealth of evidence indicates that LRRK2 is enriched at the presynaptic compartment where it regulates vesicle trafficking and neurotransmitter release. However, whether the role of LRRK2 affects neuronal networks dynamic at systems level remains unknown. Addressing this question is critical to unravel the impact of LRRK2 on brain function. Here, combining behavioral tests, electrophysiological recordings, and functional imaging, we investigated neuronal network dynamics, in vivo, in the olfactory bulb of mice carrying a null mutation in LRRK2 gene (LRRK2 knockout, LRRK2 KO, mice). We found that LRRK2 KO mice exhibit olfactory behavioral deficits. At the circuit level, the lack of LRRK2 expression results in altered gamma rhythms and odorant-evoked activity with significant impairments, while the spontaneous activity exhibited limited alterations. Overall, our data in the olfactory bulb suggest that the multifaced role of LRRK2 has a strong impact at system level when the network is engaged in active sensory processing.

## 1. Introduction

Leucine-rich repeat kinase 2 (LRRK2) is a multidomain protein pertaining to the Roco protein family [1] that has become the topic of an increasing number of studies since it was first associated with Parkinson’s disease (PD) [2,3,4]. Since then, numerous mutations of the LRRK2 gene have been identified, making LRRK2 a major genetic risk factor for inherited PD. Subsequent evidence indicates that LRRK2 mutations are also present in a small percentage of sporadic PD cases [1,5,6].

In the mammalian brain, LRRK2 is highly expressed in brain areas recipient of dopaminergic projections, such as the cerebral cortex, striatum, and the olfactory bulb (OB), both in neurons and in microglial cells [7]. At the cellular level, LRRK2 is enriched at the presynaptic terminal, where, interacting with several proteins such as Rabs [8,9], EndoA [10,11], N-ethylmaleimide sensitive factor (NSF) [5,12], orchestrates vesicle trafficking and neurotransmitter release [5,10,13,14,15]. The fact that LRRK2 operates at the synapse, as do most of the proteins encoded by genes associated with PD [6,16], prompted the hypothesis that the synapse is the first target of PD pathology. Indeed, the emerging vision is that defects in the synaptic functionality precede degeneration and neuronal loss. However, despite accumulating evidence on the action of LRRK2 at the cellular and molecular level in the presynaptic compartment, the impact of LRRK2 at systems level remains unknown. Addressing this question is critical to gain insight into the role of LRRK2 in physiological conditions.

The synapse is the elementary unit that allows the structural and functional communication among neurons, resulting in the formation of neuronal circuits. However, the impact of the synapse functionality on the information processing capability of neuronal circuits and, ultimately on the behavioral outcome, can be understood only by analyzing the synaptic activity at system level. Several theoretical and experimental evidence indicates that neuronal circuits are built to generate emergent functional states [17,18]. These states consist of recurrent patterns of activity among ensembles of neurons that serve the dynamic coordination of the neuronal responses within local and wider networks. These coactive groups of neurons (ensembles) are considered the functional units that process and encode sensory, cognitive, and motor functions [19,20].

At a different scale, oscillations, quasi-periodic and large modulations of neuronal activity present both at rest and during task execution, represent a form of highly coherent activity established to integrate neuronal responses locally and among different brain regions [21,22]. One of the brain areas where oscillations have been first identified and characterized is the OB [23]. The OB exhibits a wide range of oscillations that differ in the frequency and in the neuronal circuits that generate them. A growing body of evidence indicates that these oscillations are associated with distinct behaviors, being engaged in specific sensory and cognitive processes [24,25]. Interestingly, the OB is also one of the first brain areas involved in PD pathology [26]. Indeed, olfactory deficits precede motor impairments by several years [27]. The OB is, therefore, an interesting brain area to investigate whether and how the action of LRRK2 affects neuronal network dynamics and oscillations. To address this issue, we took advantage of a transgenic line of mice carrying a null mutation in LRRK2 (LRRK2 KO mouse). We found that, at the behavioral level, LRRK2 KO mice exhibit impairments in the olfactory discrimination test. At the circuit level, the lack of LRRK2 leads to a decrease in the amplitude of the neuronal oscillations in the 40–100 Hz band, mostly during active sniffing. Functional recording from population of neurons in the OB revealed that, upon odorant stimulation, mutant neurons have smaller and less reliable responses and exhibit a spatiotemporal profile with reduced correlation.

## 2. Materials and Methods

### 2.1. Animals

Animals were housed in filtered cages in a temperature-controlled room with a 12/12-h dark/light cycle and had access to water and food ad libitum. All procedures were conformed to the EU Directive 2010/63/EU for animal experiments and to the ARRIVE guidelines. Experimental protocols were approved by the Italian Ministry of Health.

Experiments were performed on female and male leucine-rich repeat kinase 2 knockout (LRRK2 KO) mice (C57BL/6*^-Lrrk2tm1.1Mjff^*/J, The Jackson Laboratory, Stock No: 016121) and their wild-type (WT) littermates (C57BL/6NJ background, The Jackson Laboratory, Stock No: 005304), used as internal controls, at the age of about 8 weeks. All analyses were performed blind to the genotype.

### 2.2. Habituation Dishabituation Test

The test was performed as described in [28] with minor modifications on LRRK2 KO and WT littermate control mice. During the two days preceding the experimental day, mice were put in a polycarbonate cage (20 cm × 15 cm × 13 cm) identical to the test cage for 30 min, once per day, to allow habituation to the setup.

The day of the experiment, mice were placed in the test cage for about 30 min to familiarize with the environment. After this habituation period, a piece of filtered paper (2 cm × 2 cm) with double-distilled water was presented always in the same position in the cage for 3 min. This was repeated three times with a 1-min interval (these first three trials represent the habituation trials). On the fourth trial (the dishabituation trial), filtered paper scented with eugenol (1% *v*/*v* in mineral oil) was presented into the cage. Investigation time, defined as the period of active sniffing with nasal contact with the filtered paper, was measured for each trial. To avoid odor contamination, clean cages were used for each trial. All behavioral tests were conducted during the same daytime period and were video-recorded with a CMOS camera (30 frames per sec, 2592 × 1944 pixels) for extracting the locomotion and behavioral parameters.

### 2.3. Surgical Procedure for LFP Electrode Implantation

Animals were anesthetized with a mixture of Zoletil100 (a combination of Zolazepam and Tiletamin, 1:1, 10 mg/kg; Virbac, Carros, Cedex, France) and Rompun (Xylazine 2%, 0.06 mL/kg; Bio98, Milan, Italy) and placed into a custom stereotaxic frame. A small hole was made in the skull above the OB to allow the insertion of a custom-made bipolar stainless-steel electrode into the OB at the following stereotaxic coordinates: anterior = 800 μm, lateral = 500 μm, depth = 180 μm, with respect to the intersection of the midline and the inferior cerebral vein. A small screw, used as a reference electrode, was fixed on the skull above the cerebellum. All the components were soldered to a small fingerprint connector and secured to the skull with dental cement (Paladur, AgnTho’s, Lidingö, Sweden). Mice were immediately treated with post-operative analgesia (Tramadol 10 mg/kg, Formevet, Milan, Italy). Upon waking up from the anesthesia, animals were placed in individual cages for 3 days to allow complete recovery before initiation of LFP recordings.

### 2.4. Local Field Potentials (LFP) Recordings in Freely Behaving Mice

LFP signal was amplified by a mini head-stage preamplifier (NPI electronic GmbH, Tamm, ermany) and by a 2-channels extracellular amplifier (EXT-02F NPI electronic Germany). Amplified signals were band-pass filtered (0.3–300 Hz) (EXT-02F NPI electronic, Germany) and digitized at 10 kHz (National Instruments, Austin, TX, USA). 

LFPs were recorded in LRRK2 KO and WT littermate control mice. Mice were free to move in a plexiglass arena (35 cm × 35 cm × 40 cm) placed within a Faraday cage. Before beginning the recording session, mice were habituated to the apparatus for about 30 min.

Each mouse was recorded in two distinct sessions, in two consecutive days. Each session lasted for 2 h. During the recordings in freely exploring conditions, mice were video recorded with a CMOS camera (30 frames per sec, 2592 × 1944 pixels)

Recorded videos were manually scored (MPC HC software, 1.9.5,) to identify two main behaviors: resting and exploring-sniffing. Resting indicates the behavior of an animal that is still for at least one minute. Exploring-sniffing denotes a mouse that moves around and sniffs the environment without being engaged in other tasks such as grooming. Aggregated recording datasets of 15 min were then processed for the LFP analysis.

### 2.5. Local Field Potentials Data Analysis

The raw signal acquired at 10 kHz was filtered with a 5th order band-pass Butterworth filter in 1–150 Hz range and then down-sampled at 250 Hz.

Traces for different spectral bands were obtained using filters with different band-pass ranges (4–12 Hz, 15–30 Hz, 40–70 Hz, 70–100 Hz).

Power spectral density (PSD) of LFP was computed over each behavior time interval with Welch’s method, with a Hann window with a length of 0.5 s and with 0.25 s overlap between windows, using the SciPy package for Python (https://www.scipy.org/, accessed on 1 August 2020). Spectrograms were calculated with the same parameters.

Mean power for Figure 2G was averaged over the band ranges for each mouse.

All data are expressed as mean ± SD. Mann–Whitney test or Wilcoxon test were performed in a Python environment with the SciPy package to assess the statistical significance of the data.

### 2.6. Surgical Procedures for Functional In Vivo Imaging

#### 2.6.1. Virus Injections

Animals were anesthetized with a mixture of Zoletil 100 (a combination of Zolazepam and Tiletamin, 1:1, 10 mg/kg;) and Rompun (Xylazine 2%, 0.06 mL/kg;) and placed into a stereotaxic frame. Adeno-associated viruses (AAVs) expressing the calcium indicator GCaMP6s (pAAV9.Syn.GCaMP6s.WPRE.SV40, Addgene (Watertown, MA, USA), catalog number: 100843-AAV9) were injected in each OB (500 nl of AAV vector, 70 nl/min) at the following stereotaxic coordinates: anterior = 1 mm; lateral = 500 μm; depth = 400 μm, relative to the intersection of the midline and the inferior cerebral vein. Mice were immediately treated with post-operative analgesia (Tramadol 10 mg/kg,).

#### 2.6.2. In Vivo Imaging Window Implantation

The day of the functional imaging experiment, 15–18 days after viral injection, mice were anesthetized with isoflurane (4% for induction and 1% for maintenance) and placed into a custom stereotaxic frame. Body temperature was continuously monitored and maintained at 37 °C by a thermostatic electric blanket during the entire experiment. A craniotomy (1–2 mm diameter) was made over each olfactory bulb, leaving the dura intact. The craniotomy was filled with agarose (2%) and closed with a glass window (a cover-glass 5 mm diameter, 150 μm thick; Warner Instruments, Hamden, CT, USA). The window was secured to the skull with dental cement (Paladur, AgnTho’s, Lidingö, Sweden).

### 2.7. Two-Photon Functional Imaging

In the OB, neuronal activity recordings were acquired using a two-photon microscope (Bergamo I series, Thorlabs, Newton, NJ, US). The light source, a Ti:Sapphire laser (Chameleon Ultra II, Coherent), was tuned at 920 nm to excite the genetically encoded calcium indicator GCaMP6s, and its intensity modulated using a Pockell cell (Conopitcs, Danbury, CT, USA). A main dichroic mirror (690 nm LP, Semrock, West Henrietta, NY, USA) in combination with a 525/60 nm short pass emission filter (Semrock, West Henrietta, NY, USA) directed the fluorescence photons toward the photomultiplier tube (Hamamatsu, Tokyo, Japan, H7422PA-40 model). Images were acquired at 30 frames per second, 512 × 512 pixel resolution, with a water-dipping 16x objective (Nikon, Tokyo, Japan, LWD DIC N2, N.A. 0.8) at a depth of 200–220 μm below the OB surface. Mitral cell spontaneous activity was recorded in different OB regions, imaging the same field of view (FOV) for 5 min in LRRK2 KO and WT littermate control mice. For the characterization of the sensory-induced activity, odorant stimulation was delivered by a custom olfactometer. A simple code controlling an Arduino Uno board (Arduino, Boston, Massachusset, USA) synchronized the image acquisition with the aperture of the valves of the olfactometer. For each FOV, odorant stimuli were presented in pseudo-random order (7 with the odorant mixture and 3 with mineral oil), interleaved with clean airflow. Odorant stimuli consisted of an odor mixture composed of: propionaldehyde, butyraldehyde, valeraldehyde, (+)-Carvon, citral, (−)-limonene, ethyl tiglate, eugenol (all from Merck, Danvers, Massachusset, USA). Isofluorane anesthesia at 1% was used in all the imaging experiments. All odorants were diluted 1% *v*/*v* in mineral oil.

### 2.8. Functional Imaging Analysis

Time series with functional activity were processed by means of Suite2p (in Python environment) for motion correction and semi-automatic segmentation of the cells. For each cell, the extracted fluorescence trace was filtered to reduce detection noise and slow drifts in the overall fluorescence using a 5th order digital Butterworth filter with a bandpass range from 0.005 to 0.8 Hz. The Z-score was calculated for all the traces. To quantify activation and duration of events during spontaneous activity, data were binarized based on a threshold defined as 2.5 × (σ*_FOV_* + Median*_CELL_*), where σ*_FOV_* is the standard deviation of the entire field of view and Median*_CELL_* corresponds to the median value of the intensity distribution of each cell. For each cell the amplitude, the rate, and the duration of the activity events were calculated and compared between the two groups. Correlation between amplitude and duration was assessed fitting the data to a sigmoid curve to estimate the plateau value for each strain, defined as $S\left(a\right)=D\;/\;\left(1-e^{k\left(a-a_{0}\right)}\right)$, where *D* represents the maximal duration at the higher amplitudes *a*, *k* the slew rate and *a_0_* the amplitude value corresponding to half of the duration maximum. The goodness of fit was assessed by calculating the coefficient of determination (R^2^).

Kernel density estimations (KDEs) of these data were calculated with a top-hat kernel, with a bandwidth of 0.75 using the scikit-learn package (https://scikit-learn.org/stable/index.html, accessed on 15 June 2021. The spatial structure of the activity was estimated by calculating the correlation between all the possible pairs of neurons as a function of their relative distance within the same FOV. For this purpose, Pearson’s correlation coefficient between the corresponding time series was used. For the quantification of the odor-evoked activity, a regression-based analysis was implemented, where the regression functions corresponded to the expected response of a cell to the presentation of one specific odorant. The regressors that are always zero except during the period of the stimulation, are in a number corresponding to the number of stimulation events and were convolved with an exponential decay kernel accounting for the impulsive response function of the fluorescent reporter GCaMP6s and the cell response delay (τ_ON, GCaMP6s_ = 0.11 s and τ_OFF,GCaMP6s_ = 0.52 s and response delay = 5.6 s). Using a linear regression routine available in the Scipy’s Python package (https://www.scipy.org/, accessed on 10 October 2021, the cell responses were fit to obtain a series of coefficients, each representing the amplitude of the response for the particular stimulation. Along with the amplitude of the responses, the effective duration was compared between the two groups along with the correlation between these parameters following the same procedure described above for the spontaneous activity. As for the spontaneous activity, also for the stimulated activity Pearson’s correlation between all the possible pairs of neurons was used to assess the spatial structure of the activation.

For each field with stimulation, a vector with the average response rate to each stimulus was computed, and for spontaneous activity, a similar average activity vector was calculated over 33.3 s periods.

The vectors were clustered with hierarchical clustering using the ward method, and then the relative presence of wild-type vs. mutant field of views was computed in the unbiased clusters.

### 2.9. Statistical Analysis

Statistical significance was assessed mostly using Mann–Whitney’s test and Kruskall–Wallis test where indicated.

## 3. Results

### 3.1. LRRK2 KO Mice Exhibit Olfactory Deficits

**Figure 1:**
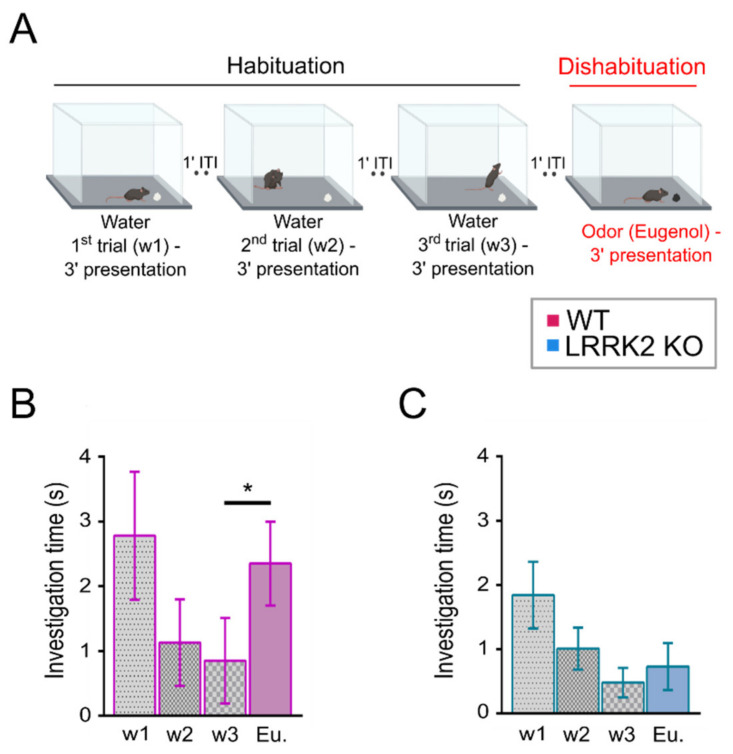
Olfactory deficits in LRRK2 KO mice. (**A**) Schematic representation of the experimental setup of the habituation–dishabituation test. The habituation phase (first three trials, black line) consisted of exposing the mice to double-distilled water (w). During the dishabituation step (fourth trial, red line), eugenol (1 mM) was presented. (**B**,**C**) Quantification of the investigation time in each trial during the habituation–dishabituation test in WT (B) and LRRK2 KO (C) mice. Statistics are calculated on mice populations. Error bars = SD. * *p* < 0.05.

To gain insight into LRRK2 biology at system level, we first analyzed the impact of a null mutation in the LRRK2 gene on the functional outcome of olfactory circuitry, performing a validated olfactory test, the habituation dishabituation task [28,29]. When WT mice were exposed to a piece of paper with water for three consecutive trials (i.e., habituation trials), they progressively spent less time sniffing above the paper, as they were habituated (1st = 2.78 ± 0.99 s; 2nd = 1.13 ± 0.67 s; 3rd = 0.85 ± 0.66 s. Mice n = 8). However, when a new slightly attractive odorant, eugenol [30], was presented on the fourth trial (dishabituation trial), WT mice spent significantly more time sniffing the new odorant with respect to the investigation time exhibited in the third trial (2.35 ± 1.83 s, *p*-value = 0.031; Figure 1B, Mice = 7). Similarly, when water was presented for three consecutive trials to LRRK2 KO mice, they were habituated (1st = 1.84 ± 0.52 s; 2nd = 1.01 ± 0.86 s; 3rd = 0.48 ± 0.41 s). However, even though LRRK2 KO locomotor activity results were similar to WT controls (32.7 ± 15.26 m and 20.1 ± 9.28 m; 2.79 ± 1.29 cm/s and 4.54 ± 2.12 cm/s, respectively; *p*-value = 0.25, Figure S1), they failed in the dishabituation trial, as they did not sniff the new odorant (eugenol) for a significantly longer amount of time in respect to the investigation time of the third trial (0.73 ± 0.67 s, *p*-value = 0.111; Figure 1C).

Altogether, these data indicated that LRRK2 KO exhibited impairment in olfactory processing.

We next examined the impact of LRRK2 mutation on neuronal circuit dynamics, namely on neuronal oscillations in the OB, the first brain area where olfactory information is processed [31]. To this end, we recorded local field potentials (LFP) in the OB in mice free to move in an arena placed in a quiet environment under dim light. In this condition, the mice spent most of the time sniffing and exploring the surrounding environment. Occasionally, they stopped and remained still from seconds to minutes. We performed spectral analysis of the LFP activity and quantified the different frequency components of the LFP signal associated with these two main behaviors, i.e., sniffing-exploring and resting.

**Figure 2:**
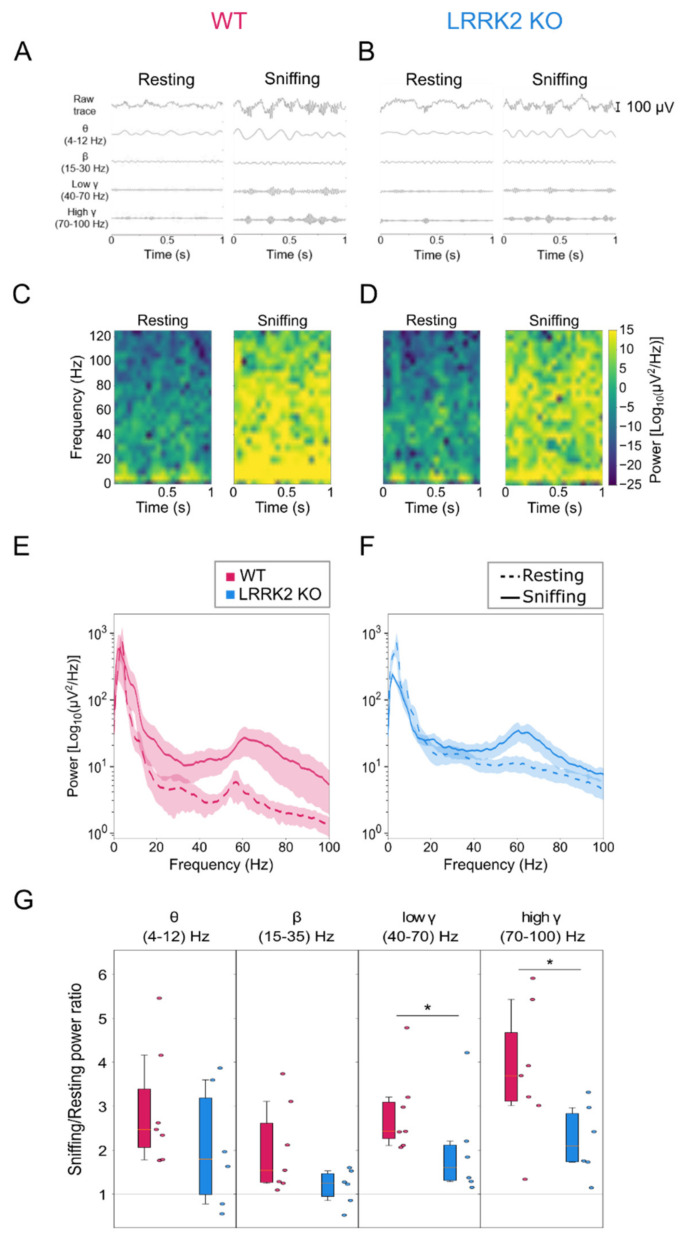
Altered network oscillations in the OB of LRRK2 KO mice. (**A**,**B**) Representative local field potential (LFP) recorded in the OB of freely behaving mice during resting (left) and sniffing (right) conditions in WT (A) and LRRK2 KO (B) mice. (**C**,**D**) Spectrogram of the LFP signal in (A) and (B), respectively. (**E**,**F**) Representative power spectrum density of recorded LFP during resting (dashed lines) and sniffing (continuous lines) behavior in one WT (E) and one LRRK2 KO (F) mouse. (**G**) Quantification of the ratio between relative powers during sniffing and resting condition, in each frequency band of oscillation, in WT and LRRK2 KO mice. Statistics are calculated on mice populations. Error bars = 5th to 95th percentile. * *p* < 0.05.

The LFP signal recorded in the OB is composed of various types of oscillations that differ in frequency (theta (1–12 Hz), beta (15–35 Hz) and gamma (low 40–70 Hz and high 70–100 Hz) and in the neuronal circuits and behavioral tasks that generate them. The whole range of these oscillations was readily observable in the LFP signal recorded in WT and LRRK2 KO mice. Bursts of gamma oscillations, which appeared more prominent during the sniffing behavior than in the resting period, were nested in the low theta oscillations (Figure 2A), as observed in previous works [24,25,32].

In WT mice, the power of the beta and gamma bands significantly increased during the sniffing behavior with respect to the resting period (in normalized power values, beta: resting = 0.70 ± 0.18; sniffing = 1.25 ± 0.20; *p*-value = 0.016. Low gamma: resting = 0.50 ± 0.08; sniffing = 1.36 ± 0.18; *p*-value = 0.016. High gamma: resting = 0.43 ± 0.17; sniffing = 1.41 ± 0.22; *p*-value = 0.016. Figure 2C–E and Figure S2; mice = 7), consistently with the role of these oscillations in odor discrimination. In addition, the sniffing period was characterized by a significant rise in the power of the theta band, which reflects the breathing rhythm during active sniffing (in normalized power values, theta: resting = 0.51 ± 0.16; sniffing = 1.33 ± 0.17; *p*-value = 0.016. Figure S2; mice = 7). These power increases were observed consistently in all the WT animals analyzed (see Figure S2) and were readily observable in the spectrogram and the power spectrum (Figure 2C–E, Figure S2).

Notably, the two genotypes displayed, on average, similar absolute power values and distribution (WT = 272 ± 121 mV^2/Hz, LRRK2 KO = 342 ± 160 mV^2/Hz, *p*-value = 0.41) in the resting and sniffing conditions.

In the LRRK2 KO mice, the transition from resting to sniffing behavior was not characterized by a univocal trend but showed an increase or a decrease in the power of theta and beta bands, depending on the animals (Figure S2). As a result of this erratic pattern of the network dynamics associated with the shift between the two behaviors, LRRK2 KO mice did not exhibit a significant rise in the power of theta and beta oscillations (in normalized power values, theta: resting = 0.75 ± 0.38; sniffing = 1.09 ± 0.20; *p*-value = 0.313; beta: resting = 0.93 ± 0.21; sniffing = 1.00 ± 0.16; *p*-value = 0.562. Figure 2D,F and Figure S1; mice = 6). The power of the gamma oscillations, however, resulted in being significantly higher during the sniffing behavior than in the resting phase in LRRK2 KO mice (in normalized power values, low gamma: resting = 0.68 ± 0.21; sniffing = 1.17 ± 0.09; *p*-value = 0.031; high gamma: resting = 0.61 ± 0.18; sniffing = 1.23 ± 0.12; *p*-value = 0.031. Figure 2D,F and Figure S2; mice = 6). We computed the ratio of the mean power in theta, beta, and gamma bands during the sniffing and the resting behavior. As shown in Figure 2G, the increase in the theta and beta power during the sniffing behavior was higher in WT than in LRRK2 KO mice, although not in a statistically significant manner (theta: WT = 2.94 ± 1.27; LRRK2 KO = 2.07 ± 1.27; *p*-value = 0.270. Beta: WT = 2.02 ± 0.95; LRRK2 KO = 1.17 ± 0.38; *p*-value = 0.111). The power of the gamma bands, however, was significantly higher during the sniffing behavior in WT than in LRRK2 KO mice. This was particularly evident for the high gamma band (low gamma: WT = 2.85 ± 0.88; LRRK2 KO = 2.01 ± 1.05; *p*-value = 0.037. High gamma: WT = 3.79 ± 1.42; LRRK2 KO = 2.22 ± 0.75; *p*-value = 0.019), which exerts a key role in odor discrimination and learning. Altogether, these data suggested an alteration of the neuronal rhythms of the OB in LRRK2 KO mice.

### 3.2. Mitral/Tufted Cells Have Similar Spontaneous Activity in WT Control and LRRK2 KO Mice

**Figure 3:**
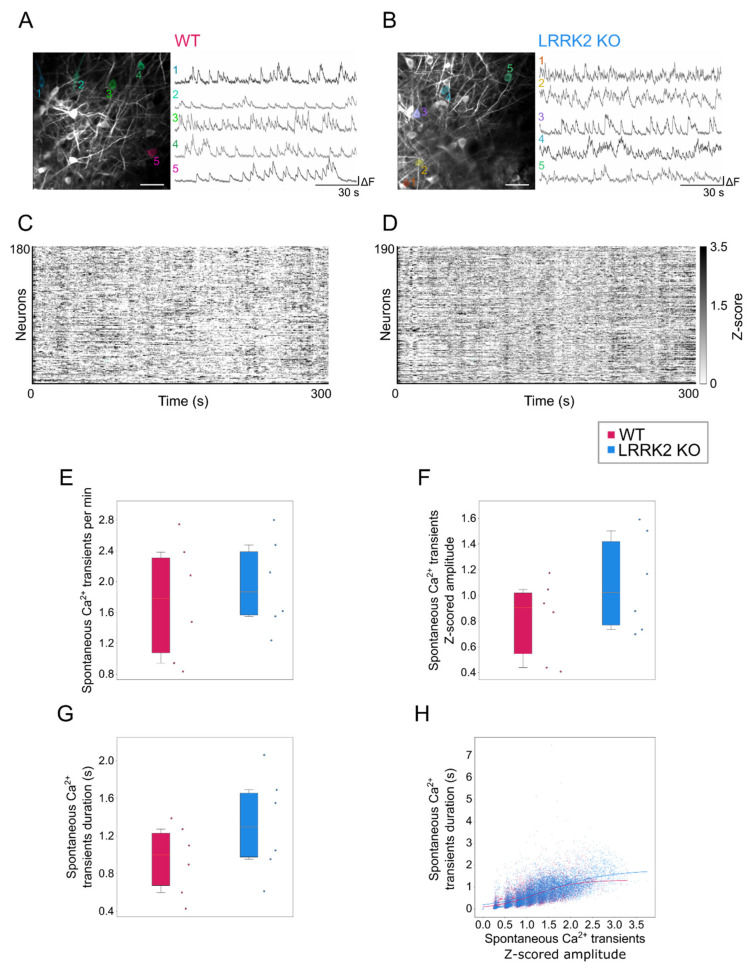
Mitral cells spontaneous activity in the OB is not affected by LRRK2 KO mutation. (**A**,**B**) Representative two-photon average projection images in vivo of GCaMP6s-expressing mitral cells (left) and spontaneous Ca^2+^ activity traces of the neurons highlighted (right) recorded in the OB of one WT (A) and one LRRK2 KO (B) mouse. Scale bars = 100 µm. (**C**,**D**) Raster plots of spontaneous Ca^2+^ activity of the mitral cells showed in A, B, respectively. (**E**–**G**) Quantification of the frequency (E), amplitude (F), and duration (G) of spontaneous Ca^2+^ transients in WT and LRRK2 KO mice. Statistics are calculated on mice populations. Error bars = 5th to 95th percentile. (**H**) Scatter plot showing the correlation between mean amplitude and mean duration of spontaneous Ca^2+^ transients for each neuron in WT and LRRK2 KO mice. The continuous lines indicate the fitted sigmoid curve (WT asymptote at duration = 1.31 s; LRRK2 KO asymptote at duration = 1.78). Statistics are calculated on cell populations.

The LFP provides a valuable measure of the level of activity and synchrony of large populations of neurons. This recording approach, however, cannot reveal the profile of the activity of single neurons pertaining to the cohort of the active cells, preventing the dissection of the cellular mechanisms underlying the abnormal oscillations we observed in KO mice. We overcame this limitation adopting two-photon functional images to record, with cellular resolution, the activity of large populations of neurons, expressing the genetically encoded calcium sensor GCaMP6s as activity reporter (see methods). Within the OB, gamma oscillations, the most affected in the KO mice, originate at the synapses between mitral and tufted cells (M/TCs) and granule cells (GCs), which are, respectively, the output neurons and the major GABAergic population of the OB [33,34]. Therefore, taking advantage of the highly organized layered structure of the OB, we recorded time series at 200–220 µm below the OB surface, corresponding to the mitral cell layer, populated by mitral cells and a subpopulation of tufted cells (M/TCs; Figure 3A,B, Videos S1 and S2).

The basal activity of M/TCs did not significantly differ between WT (mice = 6, cells = 7641) and LRRK2 KO (mice = 6, cells = 9453) mice (Figure 3A–D) when quantified as average rate of events (event rate: WT = 1.74 ± 0.36 per min, LRRK2 KO = 1.97 ± 0.27 per min, *p*-value = 0.477; Figure 3E). Although the mean amplitude of the events resulted larger (Figure 3F) and their duration (Figure 3G) longer in LRRK2 KO than in WT control mice, statistically significant differences were not observed (z-scored event amplitude WT = 0.81 ± 0.29, LRRK2 KO = 1.10 ± 0.35, *p*-value = 0.136; event duration WT = 0.95 ± 0.29 s, LRRK2 KO = 1.32 ± 0.25 s, *p*-value = 0.262).

To better evaluate the features of the spontaneous events in the two groups, we analyzed the probability distribution map of Ca^2+^ transient duration as a function of Ca^2+^ transient amplitude. We found that the two genotypes have similar maps (Figure S3). Looking at the correlation between mean amplitude and mean duration of spontaneous events in the single cells, we found that the relative distribution between these variables was equivalent in controls and LRRK2 KO mice (WT asymptote at 1.28 s duration, R^2^ = 0.465; LRRK2 KO asymptote at 1.84 s duration, R^2^ = 0.467; Figure 3H). Altogether, these data suggest that the temporal features of spontaneous events were similar in both groups of mice.

### 3.3. At Rest, Mitral/Tufted Cells Display Similar Patterns of Spatio-Temporal Correlation in WT and LRRK2 KO Mice

**Figure 4:**
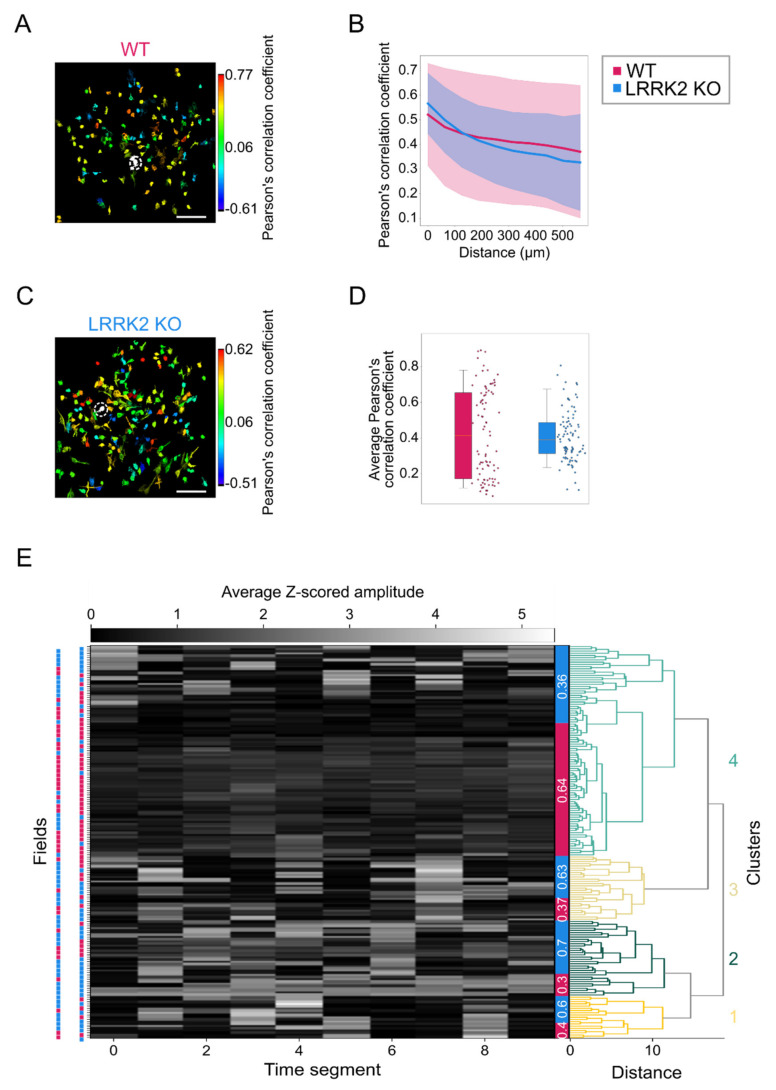
Mitral cells in the OB display a similar spatial pattern of spontaneous activity at the population level in LRRK2 KO and littermate controls. (**A**–**C**) Representative correlation maps showing the level of correlated activity between the neuron indicated by the dashed circle and every other neuron in the field of view in one WT (A) and one LRRK2 KO (C) mouse. Scale bars = 100 µm. (**B**–**D**) The spatial distribution of the average correlation between cell pairs, determined by binning their intersoma distances (56 µm per bin) in LRRK2 KO mice and littermate controls (B; shadowed area = SD), and its quantification (**D**). Error bars = 5th to 95th percentile. (**E**) Hierarchical clustering and dendrogram of the spontaneous Ca^2+^ activity, subdivided in equal temporal bins, recorded for each field of view (FOVs) from LRRK2 KO mice (cyan squares) and littermate controls (magenta squares). FOVs are grouped based on their average activity amplitude. Four clusters are visible. The proportion of FOVs for WT (magenta) and LRRK2 KO (cyan) mice in each cluster is indicated in the colored bar between the clusters and the dendrogram. Statistics are calculated on FOV populations.

We next examined the correlated activity of populations of M/TCs at rest. Analyzing the correlation between all pairs of neurons belonging to the same field of view (WT FOVs = 87, LRRK2 KO FOVs = 88; Figure 4A–D), we found that the correlation profile as a function of the distance between pairs of neurons did not differ between WT and LRRK2 KO mice (Figure 4B). Furthermore, the average correlation coefficient across FOVs was similar for the two groups (WT R^2^ = 0.43 ± 0.25, LRRK2 KO R^2^ = 0.41 ± 0.14, *p*-value = 0.409. Figure 4D).

We deepened our analysis to investigate whether FOVs showed distinct patterns of activity in control with respect to LRRK2 KO mice. To address this point, we performed hierarchical clustering analysis on all FOVs recorded evaluating the activity in 10 different 30s-long segments. This analysis revealed four main clusters composed of FOVs originating from a mixed population of controls and LRRK2 KO mice. Clusters from one to three accounted for 36% of the data and showed a ~2:1 ratio between LRRK2 KO and control mice. In contrast, cluster 4, accounting for the remaining 64%, was composed mainly by WT FOV and was characterized by a lower level of activity. All clusters exhibited a similar spatial profile of correlation as a function of the distance (Figure S4). Cluster 1, 2, 3, composed mainly by LRRK2 KO FOVs, was characterized by a higher correlation coefficient in function of the distance than cluster 4 (Figure S4A). This trend was confirmed by looking at the average correlation coefficient calculated between all pairs of neurons. In this case, cluster 4 exhibited a statistically significant lower correlation coefficient than cluster 1, 2, and 3, whose correlation coefficients were similar (Cluster 1 R^2^ = 0.54 ± 0.19, Cluster 2 R^2^ = 0.45 ± 0.16, Cluster 1 R^2^ = 0.49 ± 0.19; Cluster 4 R^2^ = 0.36 ± 0.20, *p*-value Cluster 1–4 < 0.001, *p*-value Cluster 2–4 = 0.004, *p*-value Cluster 3–4 < 0.001; Figure S4B). These results indicated that M/TCs spontaneous activity exhibited mild differences in the level of activity and in its correlation in the two genotypes.

### 3.4. Abnormal Mitral Cells Response to Odorants in LRRK2 KO Mice

The limited alterations in the spontaneous activity in M/TCs of LRRK2 KO mice suggested that the aberrant LFP activity during the sniffing/exploring phase (Figure 2) may be due to abnormal neuronal activity in response to sensory stimuli. We therefore analyzed the response to a series of odorant presentations in M/TCs by means of two-photon functional imaging. Sensory stimuli consisted of a mixture of odorants and mineral oil (used as control stimulus), delivered in pseudorandom order by a software-controlled olfactometer (Figure S5).

To analyze odor-evoked activity, we reconstructed the recorded Ca^2+^ trace employing a linear regression based on the waveforms of the stimulation of the delivered stimuli (Figure S6; see methods for details).

**Figure 5:**
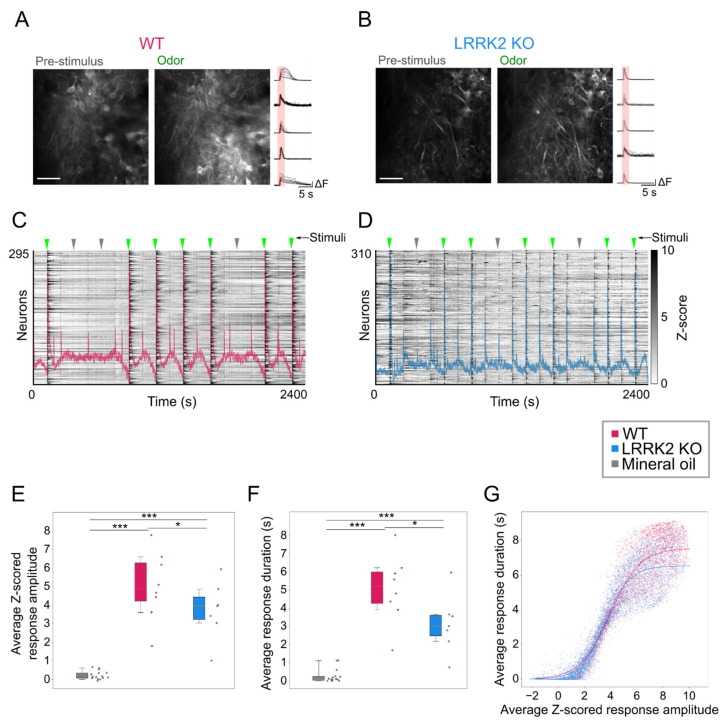
Abnormal mitral cells odor-evoked activity in the OB in LRRK2 KO mice. (**A**,**B**) Representative two-photon images in vivo of GCaMP6s-expressing mitral cells before (Pre-stimulus) and during odor stimulation (Odor) and odor-evoked Ca^2+^ responses in different neurons during repeated odor stimulation (on the right) recorded in the OB of one WT (A) and one LRRK2 KO (B) mice. Scale bars = 100 µm. (**C**,**D**) Raster plots of evoked Ca^2+^ activity of the mitral cells in (A, B) respectively; odor presentation is indicated by green arrows, mineral oil presentation by grey arrows. The mean Ca^2+^ trace related to the raster plot is superimposed. (E, F) Quantification of the amplitude (**E**) and duration (**F**) of Ca^2+^ transients driven by odor stimulation in WT and LRRK2 KO mice and in response to mineral oil stimulation. Statistics are calculated on mice populations. Error bars = 5th to 95th percentile. (**G**) Scatter plot showing the correlation between mean amplitude and mean duration of odor-evoked Ca^2+^ transients for each neuron in WT and LRRK2 KO mice. The continuous lines indicate the fitted sigmoid curve (WT asymptote at duration = 7.55 s; LRRK2 KO asymptote at duration = 6.54). Statistics are calculated on cell populations. * *p* < 0.05; *** *p* < 0.001.

Odorant stimuli elicited a prompt response in M/TCs in WT control (mice = 7, cells = 6258) and KO mice (mice = 8, cells = 6418), while both genotypes did not respond to mineral oil (used as control; Figure 5A–D, Videos S3 and S4). However, the average amplitude of the odorant-evoked events was lower and their duration shorter in LRRK2 KO mice than in WT controls (z-scored amplitude: mineral oil = 0.28 ± 0.37, WT = 5.45 ± 1.46, LRRK2 KO = 3.75 ± 1.43; Kruskal–Wallis *p*-value < 0.001, oil-WT *p*-value < 0.001, oil-KO *p*-value < 0.001, WT-KO *p*-value = 0.031. event duration: mineral oil = 0.23 ± 0.22 s, WT = 5.12 ± 1.73 s, LRRK2 KO = 3.14 ± 1.47 s; Kruskal–Wallis *p*-value < 0.001, oil-WT *p*-value < 0.001, oil-KO *p*-value < 0.001, WT-KO *p*-value = 0.028; Figure 5E–F).

The different features of the evoked responses in the two groups were confirmed by computing the probability distribution maps of the mean response duration as a function of the mean response amplitude. Using a kernel density estimation analysis, the highest probability density map related to LRRK2 KO neurons corresponded to small amplitude and short duration responses. In contrast, the probability distribution map related to WT responses covering a wider range, with the highest probability for high amplitude-long duration responses (Figure S7). The relative correlation between mean amplitude and mean duration of odorant-evoked responses was different in WT and KO mice (Figure 5G). Fitting the data with a sigmoid, we found a shift of the asymptote towards lower values for LRRK2 KO neurons with respect to controls (WT asymptote at 7.55 s duration, R^2^ = 0.852; LRRK2 KO asymptote at 6.54 s duration, R^2^ = 0.906; Figure 5G). Thus, the dynamic of the response was similar in the two mouse groups when odorants elicited low amplitude events, while LRRK2 KO showed shorter durations for larger events.

### 3.5. M/TCs Exhibit Lower Odorant Responsiveness and Correlation in LRRK2 Mutants

We then asked whether M/TCs responded reliably to repeated sensory stimulation in mutant mice. As expected, in WT mice, most M/TCs (69.84% of neurons) responded to all odorant presentations. In contrast, in LRRK2 KO mice, a consistently lower number (45.68% of neurons) of M/TCs, although not statistically significant, responded to the complete sequence of odorant-stimulations (*p*-value = 0.051. Figure S8). Furthermore, KO mice exhibited an increase in the proportion of non-responsive cells (WT: 3.65%; LRRK2 KO: 14.57%; *p*-value = 0.058), or of cells responsive to 1 stimulus (WT: 3.13%; LRRK2 KO: 10.34%; *p*-value = 0.028), or to a few stimuli (WT: 2.25%; LRRK2 KO: 5.97%; *p*-value = 0.046. Figure S8). Altogether these data indicate that in the OB of LRRK2 KO mice, M/TCs do not respond in a consistent and reliable manner to odorant stimulations.

**Figure 6:**
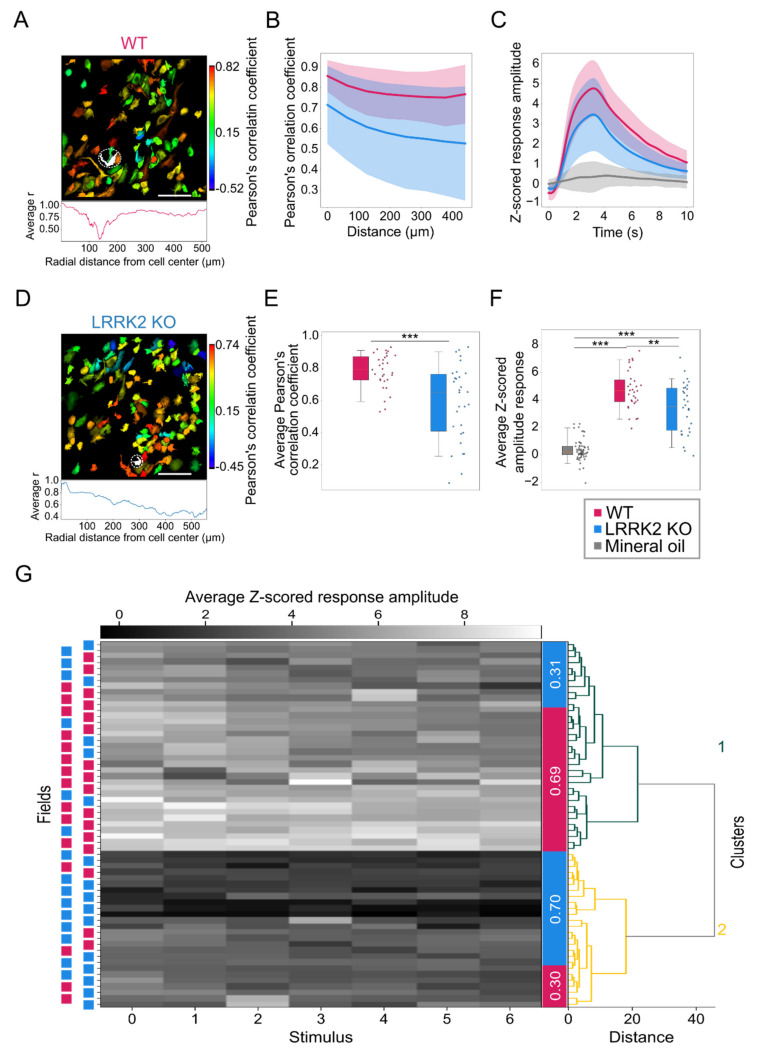
Distinct populations of mitral cells are less distinguishable during odor stimulation in the OB of LRRK2 KO mice than littermate controls. (**A**–**D**) Representative correlation maps showing the level of correlated activity between the neuron indicated by the dashed circle and every other neuron in the field of view in one WT (**A**) and one LRRK2 KO (**D**) mouse. The estimated kernel density in relation to the radial distance from the selected cell is shown below. Scale bars = 100 µm. (**B**–**E**) The spatial distribution of the average correlation between cell pairs, determined by binning their inter-soma distances (56 µm per bin) in LRRK2 KO mice and littermate controls (**B**; shadowed area = SD), and its quantification (**E**). Error bars = 5th to 95th percentile. (**C**–**F**) Average response profiles to odor stimulation in WT and LRRK2 KO mice, and to mineral oil stimulation (**C**; shadowed area = SD), and their quantification (**F**). Error bars = 5th to 95th percentile. (**G**) Hierarchical clustering and dendrogram of the odor-evoked Ca^2+^ activity, subdivided for each stimulation trial, recorded for each field of view from LRRK2 KO mice (cyan squares) and littermate controls (magenta squares). Fields of view (FOVs) are grouped based on their average correlation coefficient. Two clusters are visible. The proportion of FOVs for WT (magenta) and LRRK2 KO (cyan) mice in each cluster is indicated in the colored bar between the clusters and the dendrogram. Statistics are calculated on FOV populations. ** *p* < 0.01, *** *p* < 0.001.

Analyzing the correlation in the odor-evoked activity between all pairs of neurons in the same FOVs, we found that the spatial gradient of the correlation coefficient was higher in WT (FOVs = 31) than in LRRK2 KO mice (FOVs = 30. Figure 6A,B,D). These results were corroborated by the statistically significant difference in average correlation coefficient among all the M/T responsive cells, that was significantly higher in WT than in KO mice (WT R^2^ = 0.78 ± 0.11, LRRK2 KO R^2^ = 0.59 ± 0.23, *p*-value < 0.001; Figure 6E).

In addition, we found that the average amplitude of the M/TCs response to odorants, computed as average response for FOV, was significantly larger in WT than in LRRK2 KO mice (z-scored amplitude: Mineral oil = 0.36 ± 0.77, WT = 4.72 ± 1.41, LRRK2 KO = 3.43 ± 1.80; Kruskal–Wallis *p*-value < 0.001, WT-KO *p*-value = 0.005, oil-WT *p*-value < 0.001, oil-KO *p*-value < 0.001; Figure 6C,F).

By performing hierarchical clustering analysis on all FOVs recorded in response to odorant stimulation, we found that M/TCs evoked-activity formed two clearly separated clusters (Figure 6G). Notably, the large majority (69%) of FOVs included in cluster 1 pertained to WT mice and was characterized by stronger odorant-evoked responses. In a complementary manner, the majority (70%) of FOVs in cluster 2, characterized by a lower level of odorant-evoked activity, belonged to LRRK2 KO mice (Figure 6G).

To ascertain whether the two identified clusters recapitulate the functional features of the two genotypes, we computed the correlation coefficient as a function of the inter-soma distance among M/TCs pertaining to the different clusters. We found that the correlation coefficient was strikingly higher among neurons included in cluster 1 than in neurons within cluster 2 (Figure S9A). As a result, the average correlation coefficient was significantly higher in cluster 1 than in cluster 2 (Cluster 1 R^2^ = 0.80 ± 0.10, Cluster 2 R^2^ = 0.53 ± 0.20, test *p*-value < 0.001; Figure S9C).

Furthermore, analyzing the average response amplitude and the correlation coefficient of the identified clusters, we found that cluster 1, characterized by a higher level of neuronal activity and a prevalence of WT FOVs, contained M/TC exhibiting a response to sensory stimulation with significantly increased average amplitude (z-scored amplitude: mineral oil = 0.36 ± 0.77, Cluster 1 =5.24 ± 1.02, Cluster 2 = 2.53 ± 1.21; Kruskal–Wallis *p*-value < 0.001 C1-C2 *p*-value < 0.001, oil-C1 *p*-value < 0.001, oil-C2 *p*-value < 0.001 Figure S9B,D). Altogether, these results indicate that the two clusters recapitulated the functional features observed in the two genotypes (Figure 6).

## 4. Discussion

In this paper, we analyzed the impact of LRRK2 on the neuronal activity at system level, combining behavioral tests, electrophysiological and optical recordings of a large population of neurons in the OB of LRRK2 KO mice. The emerging picture is that the lack of LRRK2 expression significantly impairs odorant-evoked responses and the spatial correlation in the activity among the stimulus-responsive neurons, with limited effects on spontaneous neuronal activity.

At the behavioral level, LRRK2 KO mice were tested with a habituation–dishabituation test. This behavioral paradigm, in its simplicity, allows to investigate several aspects of olfactory functions. We found that LRRK2 KO mice can detect the same cue presented in subsequent trials, showing habituation. However, the absence in KO mice of a significant rise in the exploration time at the presentation of a novel odorant after the habituation, is indicative of the inability to discriminate a new scent.

This deficit in olfactory response was confirmed by the electrophysiological recordings in freely behaving mice, where we measured the OB neuronal correlates underlying the act of freely sniffing. To this end, the analysis of the LFPs recordings focused on comparing the resting and active sniffing behavior. A striking difference in neuronal network dynamics emerged during the sniffing phase in KO mice. In physiological conditions, theta oscillations largely reflect the discharge of sensory afferents from the nasal epithelium to the OB, along with low-frequency burst firing of specific OB neuronal populations, namely external tufted cells (ETCs). These oscillations track the respiratory rhythm, which is in the range 1–4 Hz during quiet-waking state and increases to 4–12 Hz during novel odor exploration and discrimination [24]. Focusing our analysis on the active exploration and sniffing behavior, we found that control animals show an increase in the power of theta in the range (4–12Hz), as expected from a higher correlated activity among the odorant-activated sensory inputs and the ETCs.

In contrast, in LRRK2 KO mice, the increase of the theta power was smaller. This difference, although not significant, points to an altered coordination in the olfactory behavior. Discriminating new odors elicits beta and gamma oscillations, whose appearance reflects the specific behavioral tasks. As in our experimental conditions, animals typically experience different environmental circumstances and behavioral states; controls show a power increase in both frequency bands. Again, LRRK2 KO mice present a more erratic trend, which results in a lower beta and gamma power increase. Interestingly, in KO mice, we observed this erratic pattern for all the frequency bands, as if sensory inputs were unable to elicit a consistent and reliable integration of neuronal responses in the OB of KO mice. With regards to gamma oscillations, it has been shown that this oscillatory activity reflects the level of synchrony of MCs firing [35,36]. Therefore, reduced gamma power indicates that MCs fire with a larger jitter relative to the gamma oscillation phase, presenting less correlated activity overall. Although we do not have a clear explanation of the strongest impairment of gamma oscillations in LRRK2 KO mice, we could speculate that this defect is due to the fact that gamma are the fastest oscillations in the OB, and this offers a workload that the LRRK2 KO networks cannot cope with. When the neuronal circuits are at rest or engaged in tasks that do not require orchestrating synaptic activity at high frequencies coherently, the network dynamics can keep up with the task, even though less reliably. However, when a more demanding performance is required, the missing role of LRRK2 in regulating the synaptic machinery significantly impairs the dynamics of neuronal networks, i.e., reduced gamma-band power.

Complementary to observations in the LFP recordings, spontaneous events recorded by means of functional imaging in single cells exhibit a similar spatio-temporal profile in the two groups, although M/TCs tend to present larger and longer responses in LRRK2 KO than in WT mice. The limited impairments of the neuronal activity at rest were further corroborated by a similar spatial profile of the correlation in neuronal activity in WT and LRRK2 KO mice. In addition, the average profile of spontaneous activity was comparable, as demonstrated also by the hierarchical clustering of all the FOVs recorded.

In contrast, odorant-evoked responses showed significant differences in the two groups of mice. Even during the online optical recordings, the FOVs in response to odorant stimulation appeared less bright in LRRK2 KO mice than in controls (Figure 5A,B). As the functional imaging relies on increases in fluorescence to track neuronal responses (i.e., putative action potentials burst) [37], the reduced change in brightness suggests a reduced response to the odorants in LRRK2 KO mice, as subsequently confirmed by the quantitative analysis. The alterations in odorant responses emerged in two different aspects. First, in KO mice, M/TCs exhibit significantly shorter and smaller odorant responses (Figure 5E,F). These results were confirmed by the distinct probability distribution maps of the response amplitude as a function of the response duration for the two genotypes. Further evidence of this trend came from the fitting of the data, characterized by a significant shift of the maximal duration of the responses towards lower values for LRRK2 KO neurons with respect to controls. Second, we found that M/TCs in the two groups show different reliability in the response to the series of successive odorant presentations. An increased number of mutant neurons responded to none or only a few scent trials, resulting in a limited number of cells that responded to the entire cycle of sensory stimulation (i.e., 7 odorant presentations). In line with these results, the recorded FOVs with lower evoked-activity originate mainly from KO mice and form a homogeneous cluster, clearly separated from the cluster with higher activity in which most FOVs are from WT mice.

This altered reliability profile was associated with a generally reduced correlated activity among all M/TCs and its spatial gradient. This lower correlation level may be linked to the reduced gamma power observed in the LFP recordings. Even though the temporal dynamics of odorant responses are complex, and it is odorant-, concentration-, and task- specific in most species, from flies to mammals, odorant presentation elicits dynamically correlated activity among the ensembles of responsive neurons [24,38,39,40,41,42,43]. This signature appears compromised in KO mice, as already observed in the LFPs recordings. As the coherence disruption in odor-encoding assemblies was reported to impair olfactory discrimination [38], we speculate that reduced correlated activity among MT/Cs may contribute to the observed impairment in discrimination for LRRK2 KO mice.

Altogether our data indicate that, at rest, the absence of LRRK2 expression does not impair the neuronal network dynamics in a significant manner. However, odor response is clearly compromised in LRRK2 KO mice. These results are compatible with functional and/or structural alterations at the level of the synapse. As evidence indicates that LRRK2 orchestrates vesicles trafficking and endocytosis in vitro [5,11,12,44,45], we favor the hypothesis that in the absence of LRRK2, these processes are slowed down. These kinds of synaptic deficits may cause mild alterations of the synaptic functionality at rest, as we observed in the LFP and functional recordings of spontaneous activity. However, they might significantly impair neurotransmission and neuronal communication during intense synaptic activity (as during high-frequency oscillations and evoked-activity), when vesicle recycling is critical to maintain proper neurotransmitter release. The lack of coordination in the molecular machinery at LRRK2 KO presynaptic terminals may therefore lead to the abnormal response profile of the single neurons and impair the activity correlation among the ensembles of the responsive cells. Along with its presynaptic role, LRRK2 was reported to be involved on the postsynaptic side in actin cytoskeleton dynamics [46] that regulates dendrite outgrowth and dendritic spine formation and consolidation. These neuronal processes exert a critical role in establishing structural connectivity among neurons. It is therefore not surprising that LRRK2 expression during development coincides with the phase of spine formation [47]. In line with the role of LRRK2 in regulating neuronal morphology via actin remodeling, it was reported that lack of LRRK2 is associated with altered spinogenesis [46,48]. These structural defects could further contribute to altered communication among neurons and altered neuronal plasticity, mostly during intense neuronal network activity. In conclusion, our work provides evidence of the physiological role of LRRK2 at system level, in coordinating the spatio-temporal pattern of neuronal activity among ensembles of neurons that are critical to process neuronal information.

## Data Availability

Data will be available upon request to the correspondent.

## Supplementary Materials

The following are available online at www.mdpi.com/article/10.3390/cells10113212/s1, Figure S1: LRRK2 KO and control mice have similar locomotor activity, Figure S2: The relative power of gamma oscillations in the OB increases both in LRRK2 KO mice and in littermate controls, Figure S3: Mitral cells spontaneous Ca^2+^ transients in the OB display similar temporal features in LRRK2 KO and littermate controls, Figure S4: Spontaneous Ca^2+^ activity in the OB of LRRK2 KO mice and littermate controls does not show clearly distinguishable activity patterns, Figure S5: Schematic representation of the multiphoton calcium imaging experimental setup during odor stimulation, Figure S6: The standard regressor of the trace of the stimuli provides a reasonable reconstruction of the recorded Ca^2+^ trace, Figure S7: MCs in the OB of LRRK2 KO mice tend to display a less reliable response to odor stimulation, Figure S8: Mitral cells temporal features of odor evoked Ca^2+^ transients in the OB are different in LRRK2 KO and littermate controls, Figure S9: Odor-evoked Ca^2+^ activity in the OB of LRRK2 KO mice and littermate controls shows clearly distinguishable activity patterns, Video S1: Representative video of functional spontaneous activity recorded in the OB of a GCaMP6s-expressing WT mouse, Video S2: Representative video of functional spontaneous activity recorded in the OB of a GCaMP6s-expressing LRRK2 KO mouse, Video S3: Representative video of functional activity recorded in the OB of a GCaMP6s-expressing WT mouse in response to odorant presentation, Video S4: Representative video of functional activity recorded in the OB of a GCaMP6s-expressing LRRK2 KO mouse in response to odorant presentation.
