# Supplementary material for: Aberrant Patterns of Sensory-Evoked Activity in the Olfactory Bulb of LRRK2 Knockout Mice
Source: Cells. 2021 Nov 17;10(11):3212. doi: 10.3390/cells10113212 (PMC8622670; doi:10.3390/cells10113212)
Supplement: Supplementary file 1 [file cells-10-03212-s001.zip › cells-1424604 - suppl. materials/cells-1424604 - suppl. material.pdf]

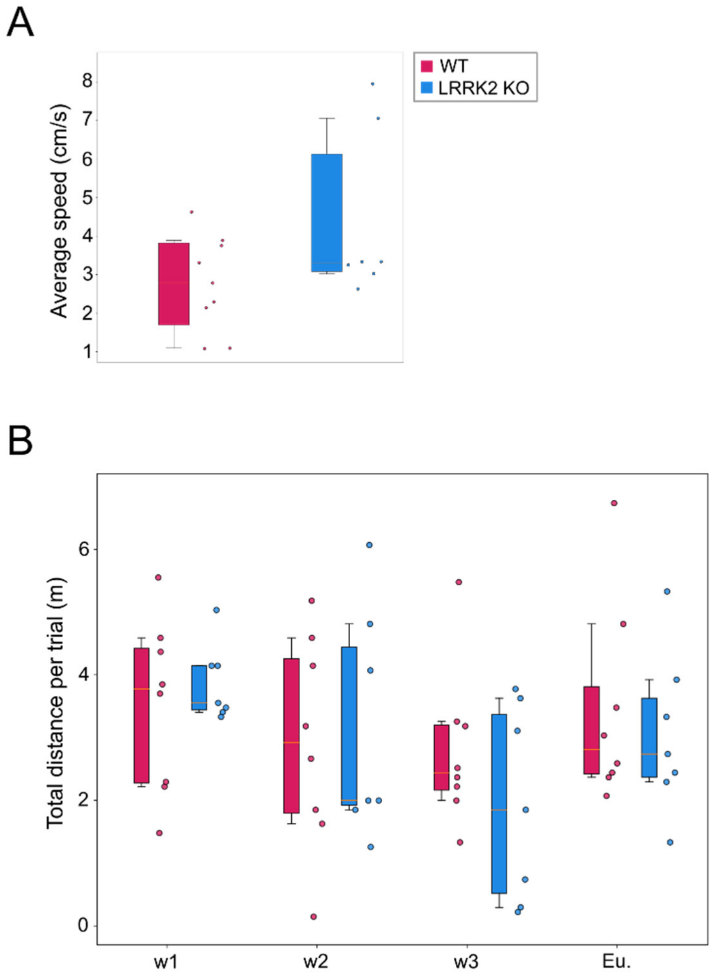

Figure S1. LRRK2 KO and control mice have similar locomotor activity. (**A**) Quantification of WT and LRRK2 KO mice's locomotion expressed as average speed reached by animals during explorative behavior. (**B**) Quantification of the distance travelled by WT and LRRK2 KO mice in each trial of the habituation-dishabituation test showed in Figure 1A,B. Error bars = 5th to 95th percentile.

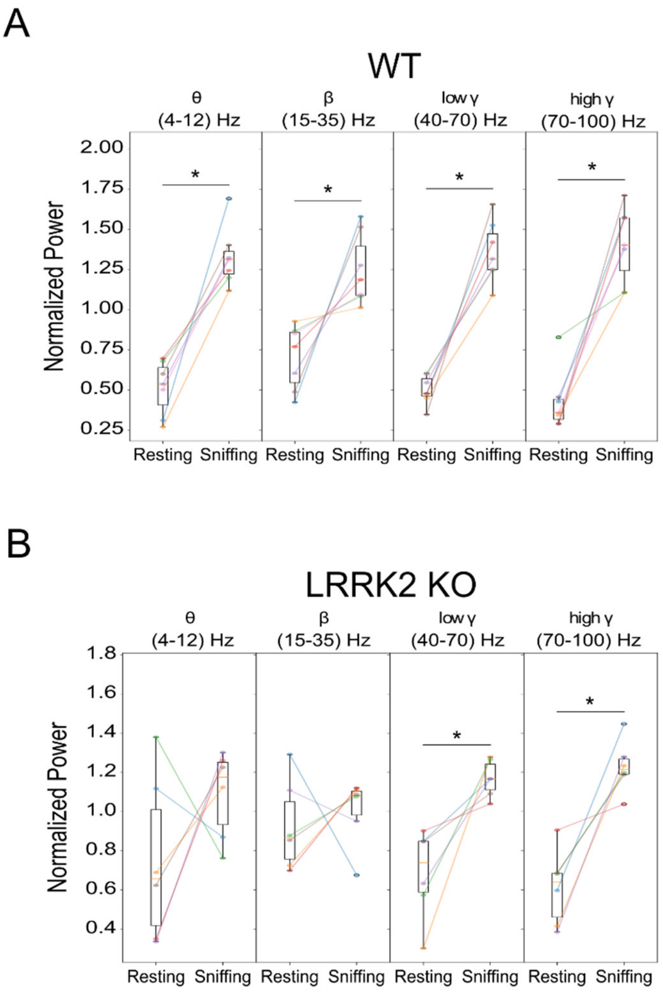

Figure S2. The relative power of gamma oscillations in the OB increases both in LRRK2 KO mice and in littermate controls. (**A**,**B**) Quantification of the different frequency bands of oscillation relative power increase in WT (A) and LRRK2 KO (B) mice during sniffing behavior compared to resting behavior. Statistics are calculated on mice populations. Error bars = 5th to 95th percentile. * $p < 0.05$.

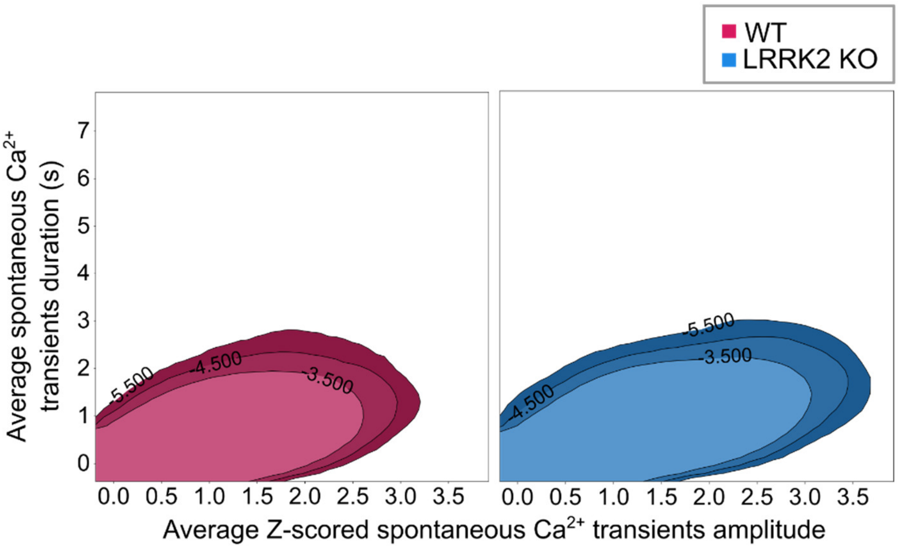

Figure S3. Mitral cells spontaneous Ca^{2+} transients in the OB display similar temporal features in LRRK2 KO and littermate controls. Kernel density estimation map of the scatter plot in Figure 3 A showing the probability distribution of the average spontaneous activity in relation to amplitude and duration of Ca^{2+} transients in WT (left) and LRRK2 KO (right) mice. Statistics are calculated on cells populations.

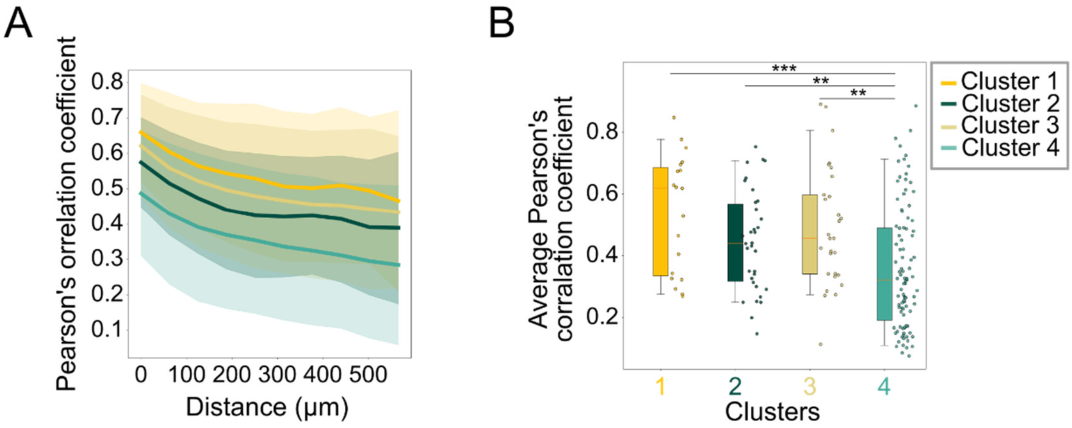

Figure S4. Spontaneous Ca^{2+} activity in the OB of LRRK2 KO mice and littermate controls does not show clearly distinguishable activity patterns. (**A**,**B**) The spatial distribution of the average correlation between cell pairs, determined by binning their intersoma distances (56 µm per bin) for each cluster obtained with the hierarchical clustering approach shown in Figure 4E (A; shadowed area = SD), and its quantification (B). Statistics are calculated on fields of view (FOVs) populations. ** $p < 0.01$, *** $p < 0.001$.

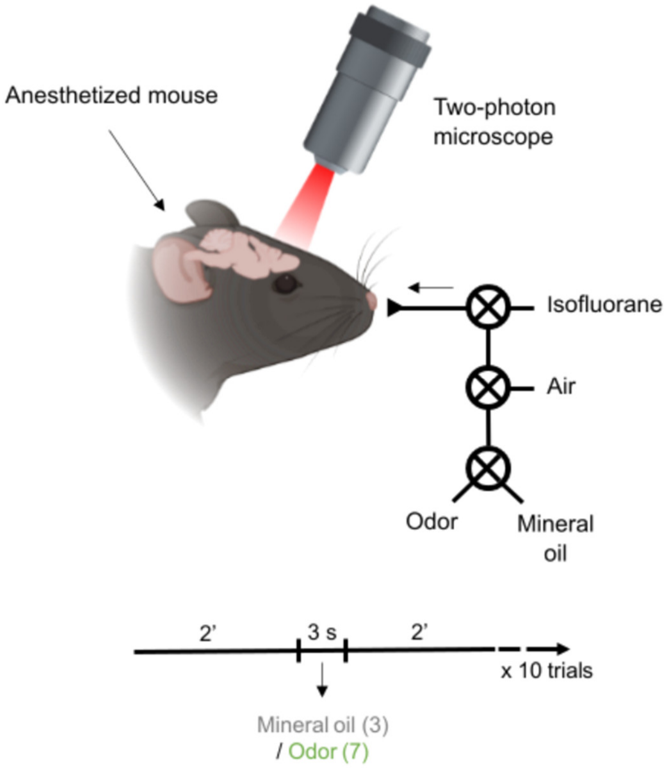

Figure S5. Schematic representation of the multiphoton calcium imaging experimental setup during odor stimulation. Mice are under continuous isofluorane anesthesia. The imaging consisted of 10 consecutive trials of stimulation (seven odor mix and three mineral oil puffs) of 3 seconds each. Clean air was continuously delivered during the two minutes of baseline and the four minutes of the interstimulus period.

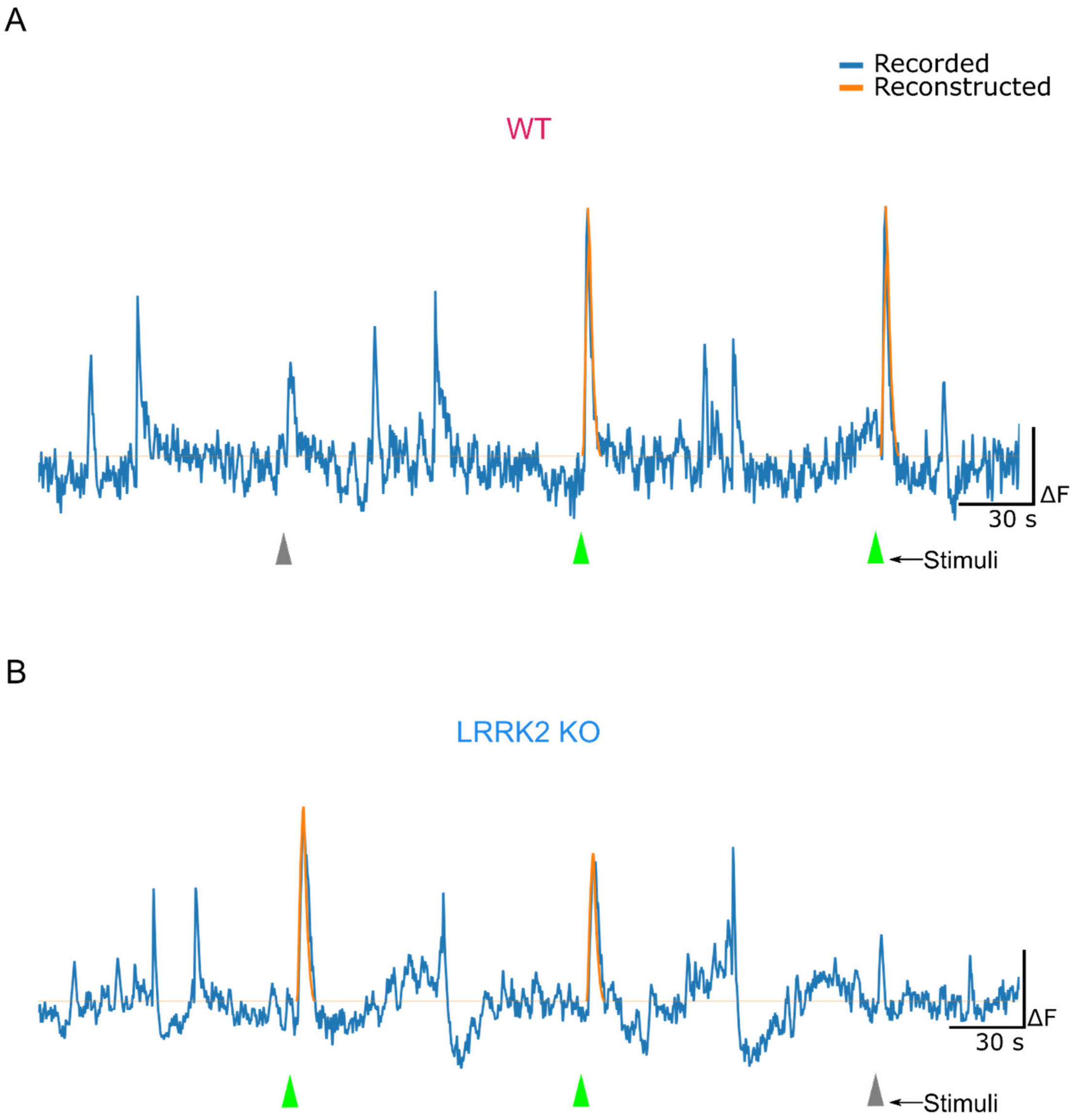

Figure S6. The standard regressor of the trace of the stimuli provides a reasonable reconstruction of the recorded Ca^{2+} trace. (**A**,**B**) Representative recorded odor-evoked Ca^{2+} traces (blue) and their respective reconstructed traces (orange) in WT (A) and LRRK2 KO (B) mice. The reconstructed trace is obtained by multiplying the standard regressor of the trace of the stimuli delivered by its coefficient and convolving it with the original trace. To better visualize the fitting of the reconstructed trace upon the recorded one, traces are cut around three consecutive stimuli (one mineral oil and two odors), which are indicated by the arrows (odor: green; mineral oil: grey).

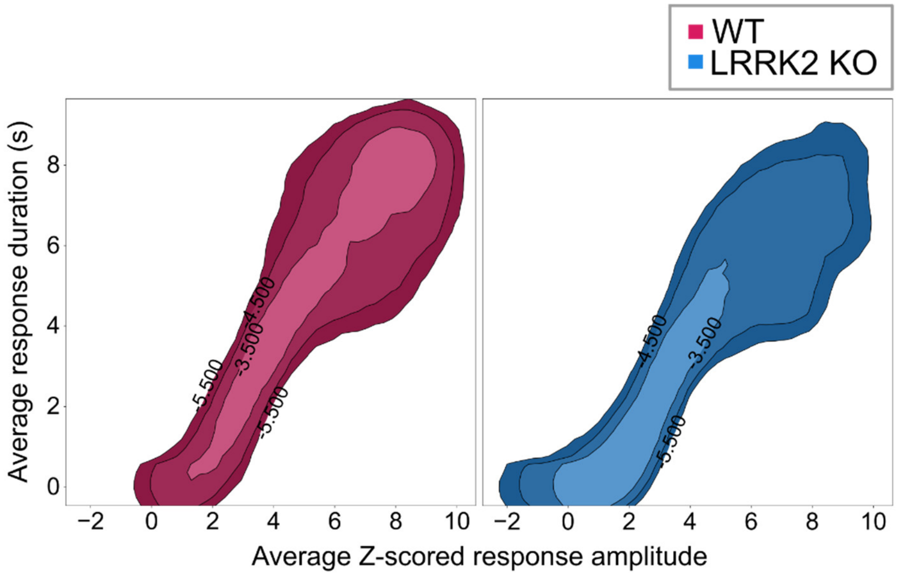

Figure S7. Mitral cells' temporal features of odor-evoked Ca^{2+} transients in the OB are different in LRRK2 KO and littermate controls. Kernel density estimation map of the scatter plot in Figure 5G, showing the probability distribution of the average evoked activity in relation to amplitude and duration of Ca^{2+} transients in WT (left) and LRRK2 KO (right) mice. Statistics are calculated on cells populations.

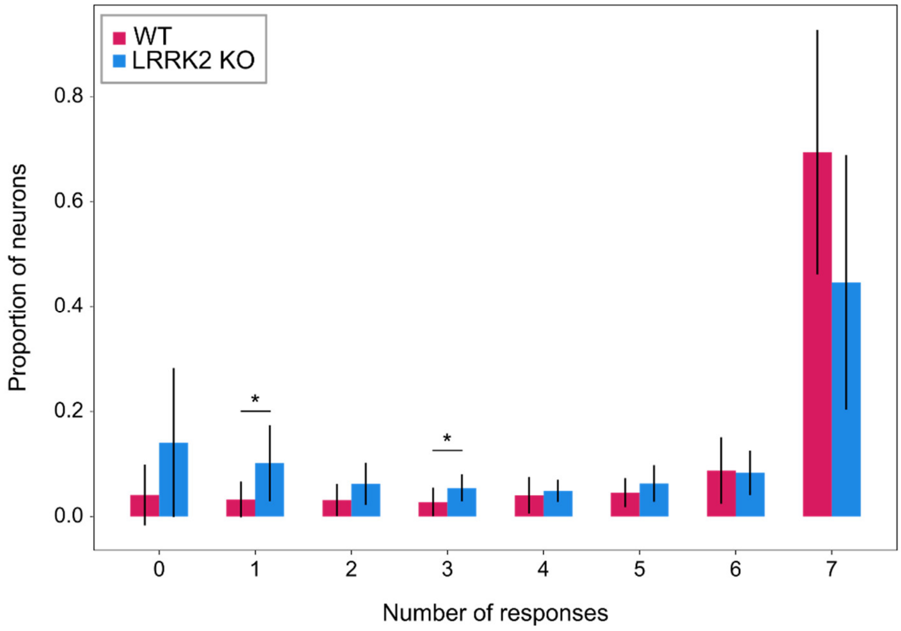

Figure S8. MCs in the OB of LRRK2 KO mice tend to display a less reliable response to odor stimulation. (A) Histogram showing the proportion of MCs responding to different odor stimuli in WT and LRRK2 KO mice. Statistics are calculated on mice populations. Error bars = SD. * $p < 0.05$.

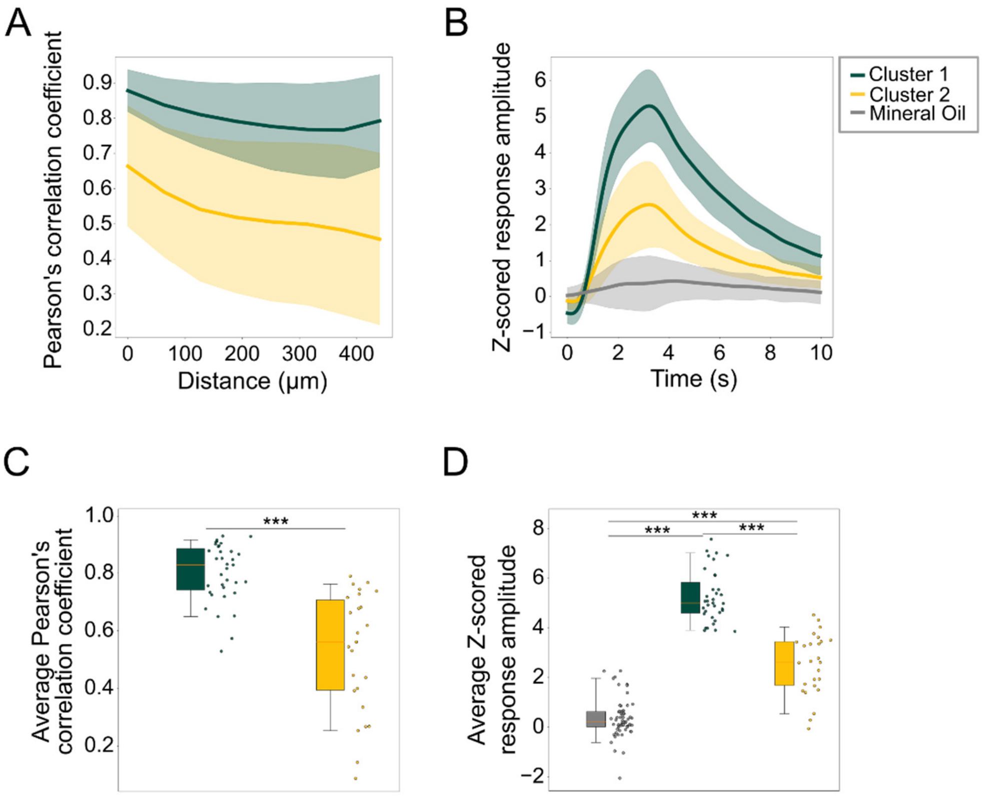

Figure S9. Odor-evoked Ca^{2+} activity in the OB of LRRK2 KO mice and littermate controls shows clearly distinguishable activity patterns. (**A**,**C**) The spatial distribution of the average correlation between cell pairs, determined by binning their intersoma distances (56 µm per bin) for each cluster obtained with the hierarchical clustering approach shown in Figure 6G (A; shadowed area = SD), and its quantification (C). (**B**,**D**) Average response profiles to odor stimulation for each cluster obtained with the hierarchical clustering approach shown in Figure 6G, and to mineral oil stimulation (B; shadowed area = SD) and their quantification (D). Error bars = 5th to 95th percentile. Statistics are calculated on fields of view (FOVs) populations. *** $p < 0.001$.

Video S1. Representative video of functional spontaneous activity recorded in the OB of a GCaMP6s-expressing WT mouse.

Video S2. Representative video of functional spontaneous activity recorded in the OB of a GCaMP6s-expressing LRRK2 KO mouse.

Video S3. Representative video of functional activity recorded in the OB of a GCaMP6s-expressing WT mouse in response to odorant presentation.

Video S4. Representative video of functional activity recorded in the OB of a GCaMP6s-expressing LRRK2 KO mouse in response to odorant presentation.
